# Trends in clinical features, postoperative outcomes, and long-term survival for gastric cancer: a Western experience with 1,278 patients over 30 years

**DOI:** 10.1186/1477-7819-12-217

**Published:** 2014-07-16

**Authors:** Fausto Rosa, Sergio Alfieri, Antonio Pio Tortorelli, Claudio Fiorillo, Guido Costamagna, Giovanni Battista Doglietto

**Affiliations:** 1 Department of Digestive Surgery, Catholic University, “A. Gemelli” Hospital, Largo A. Gemelli, 8, Rome 00168, Italy; 2 Department of Digestive Endoscopy, Catholic University, “A. Gemelli” Hospital, Rome, Italy

**Keywords:** Gastric cancer, Surgery, Long-term survival

## Abstract

**Background:**

The aim of the present study was to identify temporal trends in long-term survival and postoperative outcomes and to analyze prognostic factors influencing the prognosis of patients with gastric cancer (GC) treated in a 30-year interval in a tertiary referral Western institution.

**Methods:**

Between January 1980 and December 2010, 1,278 patients who were diagnosed with GC at the Digestive Surgery Department, Catholic University of Rome, Italy, were identified. Among them, 936 patients underwent surgical resection and were included in the analysis.

**Results:**

Over time there was a significant improvement in postoperative outcomes. Morbidity and mortality rates decreased to 19.4% and 1.6%, respectively, in the last decade. By contrast, the multivisceral resection rate steadily increased from 12.7% to 29.6%. The overall five-year survival rate steadily increased over time, reaching 51% in the last decade, and 64.5% for R0 resections. Multivariate analysis showed a higher probability of overall survival for early stages (I and II), extended lymphadenectomy, and R0 resections.

**Conclusions:**

Over three decades there was a significant improvement in perioperative and postoperative care and a steady increase in overall survival.

## Summary

The aim of the present study was to identify, over a 30-year period, temporal trends in long-term survival and postoperative outcomes and to analyze prognostic factors influencing the prognosis of gastric cancer (GC) patients in a tertiary referral Western institution.

## Background

Despite a major decline in incidence and mortality, gastric cancer (GC) remains an important public health burden worldwide. Nearly one million (988,000) new cases of stomach cancer were recorded in 2008, accounting for 7.8% of all cancer cases. At the same time, 736,000 people died from gastric cancer, representing 9.7% of all cancer deaths. Hence, GC is the fourth most commonly occurring cancer after cancer of the lung, breast, and colon-rectum, and the second most common cancer-related cause of death after lung cancer [1].

Over the past several decades, the epidemiologic profile of GC has changed dramatically. Although the incidence of GC is decreasing, the incidence of esophago-gastric junction (EGJ) cancer is increasing [2].

Survival after surgery of GC has been deeply studied in multiple series with patients stratified by stage of disease, Lauren tumor type, tumor location, time period, and administration of adjuvant therapy. All studies uniformly show an association between stage and survival. The Lauren classification significantly correlates with survival, in the fact that intestinal-type tumors are associated with longer survival than are diffuse-type tumors [3]. With regard to tumor location, some series show a significant association with survival, with GC having an improved prognosis over EGJ cancer [2,3].

Screening and the widespread use of endoscopy have been shown to be effective in the early diagnosis of cancer [4,5]. Preoperative staging techniques have improved with time, allowing better selection of patients, while operative techniques and perioperative management have also evolved, leading to decreased morbidity and mortality [6,7]. Every day, new information emerges in the cancer field that has the potential to change cancer care.

In the light of these findings, we overviewed 1,278 patients with gastric cancer over a 30-year period. The aim of the present study was to identify temporal trends in long-term survival and postoperative outcomes and to analyze prognostic factors influencing the prognosis of GC patients in a tertiary referral Western institution.

## Methods

A review of the prospective database of GC at the Digestive Surgery Unit, Department of Surgery, Catholic University, Rome, Italy, identified 1,278 patients who were diagnosed with GC between January 1980 and December 2010. Institutional review board approval was obtained before review of the patients’ medical records. Catholic University Institutional Review Board approved the study.

In order to demonstrate changes over time, the time trends were examined by comparing three time periods (1980-1989, 1990-1999, and 2000-2010). These three periods were chosen to achieve a balance of sufficient sample number and adequate follow-up.

Informed consent regarding surgical treatment, follow-up, and data management for research studies was obtained from all included patients.

We recorded hospital morbidity and mortality, type of treatment, histologic type according to Lauren [8], and demographic characteristics, tumor size, location, and gross appearance according to Borrmann [9]. The disease was staged according to the 7th Edition of the American Joint Committee on Cancer and the International Union Against Cancer Staging System (UICC) [10]. Based on categories established by the Japanese Gastric Cancer Association [11], the regional extent of nodal involvement after radical procedures was also recorded. Tumors located proximally were classified according to Siewert and Stein [12]. Only Siewert type III tumors were included in the analysis.

All patients with potentially curable lesions were treated by gastrectomy and D2 lymphadenectomy. Patients with stage IV disease and noncurable lesions (distant metastases, peritoneal carcinomatosis, and N4 nodal involvement) at the preoperative evaluation were either treated by palliative gastrectomy and perigastric (D1) lymphadenectomy with the intent to control specific symptoms (bleeding and/or obstruction) and to obtain survival advantage or, in the last period, patients with bulky nodal disease received perioperative chemotherapy.

For tumors located in the middle and lower thirds of the stomach, a subtotal gastrectomy was generally preferred, provided that an adequate resection margin was maintained. Gastrectomy was always completed by removal of the greater omentum and perigastric lymph nodes; extended lymphadenectomy was performed according to the criteria subsequently described by the Japanese Gastric Cancer Association [11].

The reconstruction of digestive continuity after total gastrectomy was previously described [13]. After subtotal distal gastrectomy, a gastrojejunostomy according to the Billroth II operation was usually performed. In case of upper polar resection (with or without transhiatal/abdominothoracic esophagectomy), an esophagogastrostomy was performed, manually until the early 1980s and later using a 25-mm mechanical circular stapler with a row of external seromuscular sutures with interrupted absorbable stitches.

Resection was stated as potentially curative (R0 according to the UICC) if macro- and microscopically no tumor was left following surgery [10].

Extensive surgery (multiorgan resection) because of suspicion of direct tumor invasion was defined as combined resection of adjacent organs (spleen, left pancreas, liver, colon, adrenal gland, diaphragm, abdominal wall, and small intestine).

At the end of the operation, the surgeon resected all lymph nodes from the surgical specimen and identified their distribution and tumor location according to the classification subsequently described by the Japanese Gastric Cancer Association [11].

The patients were monitored for 30-day postoperative complications and mortality.

As far as combined treatments are concerned, 28 patients, all in the third period (2000-2010), received perioperative neoadjuvant therapy according to the MRC Adjuvant Gastric Infusional Chemotherapy (MAGIC) protocol [14].

Three hundred sixty-one patients received postoperative adjuvant therapy. Adjuvant regimens were highly varied over the 30 years spanned by this series. As previously reported [13], the decision to administer adjuvant chemotherapy was made by medical oncologists. This resulted in heterogeneous indications for chemotherapy, treatment protocols, and number of cycles performed.

Therefore, details of adjuvant and neoadjuvant chemotherapy were not considered for statistical analysis.

### Statistical analysis

All clinical and pathological data were prospectively stored in a GC database and retrospectively evaluated for this study.

Patient status was investigated by follow-up examination or by telephone contact. Complete follow-up information was obtained as of 30 September 2012. The complete follow-up rates from 1980-1989, 1990-1999, and 2000-2010 were 95%, 97%, and 98%, respectively.

Statistical analysis was performed using commercially available software (SPSS® for Windows version 20.0; Chicago, IL). Results are given as mean (SD). The statistical significance of the difference between mean values was evaluated using the Student’s *t*-test. All tests were two tailed. Categorical variables were assessed by the Pearson’s chi-squared test. Multivariable analysis was undertaken using the Cox proportional hazards model. Survival curves were estimated using the Kaplan-Meier method, and differences between groups were evaluated using the log-rank test. *P* < 0.05 was considered statistically significant.

## Results

Among 1,278 patients, 90 patients were not resected and were addressed to palliative therapies, 147 were found on exploration to have irresectable disease, and 105 received just a bypass procedure. Gastrectomy was possible in the remaining 936 patients, resulting in an overall resectability rate of 73.2%.

All 936 patients were included in the analysis: n = 275 in the period 1980-1989, n = 239 in the period 1990-1999, and n = 422 in the period 2000-2010.

The different types of surgical procedures are reported in Table 1. After a decline in observation in the 1990s for GC, in the period 2000-2010 there was an exponential increase.

**Table 1:** **Patients observed (1980-2010*)***

**Surgical procedure**	**1980-1989 (n = 440)**	**1990-1999 (n = 320)**	**2000-2010 (n = 518)**	**1980-2010 (n = 1,278)**	**Resected (936)**
Total gastrectomy	128 (29.1)	140 (43.8)	182 (35.1)	**450 (35.2)**	
Subtotal distal gastrectomy	139 (31.6)	90 (28.1)	204 (39.4)	**433 (33.9)**	
Total degastro-gastrectomy	5 (1.1)	9 (2.8)	21 (4)	**35 (2.7)**	
Upper polar resection	3 (0.7)	0 (0)	15 (2.9)	**18 (1.4)**	
Bypass procedure	69 (15.7)	19 (5.9)	17 (3.3)	105 (8.2)	
Exploratory laparotomy	50 (11.3)	34 (10.6)	63 (12.2)	147 (11.5)	
No surgery	46 (10.6)	28 (8.8)	16 (3.1)	90 (7.1)	

Values in bold are percentages.

The patient characteristics are given in Table 2.

**Table 2:** Details of patient and tumor characteristics in 936 patients undergoing tumor resection

	**All patients (n = 936)**	**1980-1989 (n = 275)**	**1990-1999 (n = 239)**	**2000-2010 (n = 422)**	P*
**Gender**					
Male	581 (62)	176 (64)	150 (62.7)	255 (60.4)	0.63
Female	355 (38)	99 (36)	89 (37.3)	167 (39.6)	
**Age (years)**					
<65	503 (53.7)	153 (55.6)	129 (54)	221 (52.4)	0.70
≥65	433 (46.3)	122 (44.4)	110 (46)	201 (47.6)	
**Tumor location**					
Lower third	405 (43.2)	131 (47.6)	102 (42.7)	172 (40.7)	0.31
Middle third	337 (36)	90 (32.7)	82 (34.3)	165 (39)	
Upper third	179 (19.1)	52 (18.9)	49 (20.5)	78 (18.5)	
Whole stomach	15 (1.6)	2 (0.2)	6 (2.5)	7 (1.6)	
**Tumor stage**					
I	255 (27.2)	48 (17.4)	68 (28.4)	139 (33)	
II	197 (21)	70 (25.4)	32 (13.4)	95 (22.5)	<0.001
III	319 (34)	97 (35.3)	88 (36.8)	134 (31.7)	
IV	165 (17.6)	60 (21.8)	51 (21.3)	54 (12.8)	
**Lauren classification**					
Diffuse	402 (42.9)	138 (50.2)	105 (43.9)	159 (37.6)	
Intestinal	449 (48)	109 (39.6)	126 (52.7)	214 (50.8)	<0.001
Indeterminate	85 (9.1)	28 (10.2)	8 (3.4)	49 (11.6)	
**Borrmann classification**					
I	192 (20.5)	59 (21.5)	49 (20.5)	84 (19.9)	0.83
II	316 (33.8)	80 (29.1)	78 (32.6)	158 (37.4)	
III	276 (29.5)	92 (33.4)	83 (34.7)	101 (23.9)	
IV	137 (14.6)	38 (13.8)	27 (11.4)	72 (17.1)	
Undetermined	15 (1.6)	6 (2.2)	2 (0.8)	7 (1.7)	
**Tumor size, mean (SD), cm**	4.9 ± 3.6	6.5 ± 4.1	4.4 ± 2.7	4.2 ± 2.8	<0.001
**Distant metastasis**	74 (7.9)	20 (7.3)	17 (7.1)	37 (8.8)	0.635

Values in parentheses are percentages. *Two-tailed Pearson’s chi-squared test.

As expected, some characteristics differed between the three treatment periods.

The most common tumor location was the lower third in 405 (43.2%) patients for all three periods, followed by the middle third in 337 (36%), the upper third in 179 (19.1%), and the whole stomach in 15 (1.6%).

Histologic evaluation was available for all the resected specimens. Most carcinomas showed intestinal type differentiation (47.9% for the three periods) with a significant difference between the first and the third decade (39.6% versus 50.8%; *P* < 0.001).

In our experience, stage I GC significantly increased over time with a significant reduction of stages III and IV (*P* < 0.001).

Surgical characteristics according to treatment period are reported in Table 3.

**Table 3:** Surgical characteristics according to treatment period

	**Whole series (n = 936)**	**1980-1989 (n = 275)**	**1990-1999 (n = 239)**	**2000-2010 (n = 422)**	P*
**Extent of resection**					
Total gastrectomy	450 (48)	128 (46.6)	140 (58.6)	182 (43.1)	<0.001
Subtotal distal gastrectomy	433 (46.3)	139 (50.5)	90 (37.6)	204 (48.4)	
Total degastro-gastrectomy	35 (3.8)	5 (1.8)	9 (3.8)	21 (5)	
Upper polar resection	18 (1.9)	3 (1.1)	0	15 (3.5)	
**Margin status**					
R0	723 (77.2)	213 (77.5)	178 (74.5)	336 (79.6)	0.689
R1/2	213 (22.8)	62 (22.5)	61 (25.5)	90 (21.4)	
**Multivisceral resections**	195 (21)	35 (12.7)	37 (15.5)	123 (29.6)	<0.001
**Lymph nodes retrieved**					
<15	225 (24)	114 (41.5)	31 (13)	71 (16.8)	<0.001
15-30	327 (35)	84 (30.5)	66 (27.6)	157 (37.2)	
≥30	384 (41)	77 (28)	142 (59.4)	194 (46)	
Perioperative morbidity**	230 (25.3)	74 (26.9)	72 (30.1)	82 (19.4)	0.004
Anastomotic leak	39 (4.2)	8 (2.9)	15 (6.3)	16 (3.8)	
Intra-abdominal abscess	27 (2.9)	11 (4)	11 (4.6)	5 (1.2)	
Abdominal wound dehiscence	4 (0.4)	2 (0.7)	1 (0.4)	1 (0.2)	
Wound infection	11 (1.2)	8 (2.9)	2 (0.8)	1 (0.2)	
Duodenal stump dehiscence	6 (0.6)	3 (1)	0	3 (0.7)	
Pneumonia	31 (3.3)	13 (4.7)	8 (3.3)	10 (2.4)	
Pulmonary embolism	5 (0.5)	3 (1)	1 (0.4)	1 (0.2)	
Hemorrhage	26 (2.8)	1 (0.4)	10 (4.2)	15 (3.5)	
Pancreatic fistula	10 (1)	2 (0.7)	5 (2)	3 (0.7)	
Intestinal obstruction	3 (0.3)	2 (0.7)	0	1 (0.2)	
Melena	3 (0.3)	1 (0.4)	1 (0.4)	1 (0.2)	
Other	69 (7.4)	23 (8.4)	18 (7.5)	28 (6.6)	
**Perioperative mortality**	33 (3.7)	17 (6.2)	10 (4.2)	6 (1.6)	0.009

Values in parentheses are percentages. *Two-tailed Pearson’s chi-squared test. **The total number of patients having complications is less than the sum of patients having individual complications because some patients had more than one complication.

The most common operations were total gastrectomy (48%), subtotal distal gastrectomy (46.3%), total degastro-gastrectomy (3.8%), and proximal resections (upper polar resection with or without transhiatal/abdominothoracic esophagectomy) (1.9%).

Total gastrectomy was the most common procedure in the second decade, whereas subtotal distal gastrectomy was most common in the first and third decades.

Overall, 723 patients were categorized as R0 resections (77.2%). There were no significant differences in these data concerning the three time intervals.

Noncurative resections were performed in 213 patients (22.8%).

Overall morbidity and mortality were 25.3% and 3.7%, respectively.

Over time, multivisceral resections and lymph nodes retrieved constantly increased over the three periods, but did not result in higher morbidity and mortality.

Factors affecting five-year overall survival according to univariate and multivariate analysis are reported in Tables 4 and 5, respectively.

**Table 4:** Factors affecting five-year overall survival according to univariate analysis

		**Five-year overall survival (%)**			
	**All patients (n = 936)**	**1980-1989**	**1990-1999**	**2000-2010**	P*
**Gender**					
Male	581 (62)	39.6	46.6	51.3	<0.001
Female	355 (38)	37.8	52.2	53.4	0.02
**Age**					
<65	503 (53.7)	45.8	53	55	0.001
≥65	433 (46.3)	31.1	42	47	0.004
**Tumor location**					
Lower third	405 (43.3)	40.2	50.5	54.5	0.003
Middle third	337 (36)	46.2	59.5	57.9	0.03
Upper third	179 (19.1)	27	30.6	38.1	0.03
Whole stomach	15 (1.6)	18	25.3	33.4	0.027
**Lauren classification**					
Diffuse	402 (42.9)	32.8	47.5	47	0.003
Intestinal	449 (48)	48.8	48.1	59.1	0.017
Indeterminate	85 (9.1)	37	34.3	35	0.18
**Tumor stage**					
I	255 (27.2)	77	74.9	76.8	0.035
II	197 (21.1)	51.2	61.6	67.1	0.075
III	319 (34.1)	28.5	31.4	38.7	0.024
IV	165 (17.6)	11.9	10.6	14.6	0.320
**Type of gastrectomy**					
Total gastrectomy	450 (48)	43.9	46.5	50.8	0.12
Distal subtotal gastrectomy	433 (46.2)	45.9	51.6	57.6	<0.001
**Margin status**					
R0	723 (77.2)	51	60.5	64.5	<0.001
R1/2	213 (22.8)	20	23	26	0.158
**Adjuvant therapy**					
Yes	361 (39)	27.2	29.6	35.2	0.138
No	571 (61)	56.1	57	55.3	0.286
**Multivisceral resections**	195 (21)	24.9	31.7	49.6	<0.001
**Lymph nodes retrieved**					
<15	225 (24)	22.4	30.4	39.2	<0.001
15-30	327 (35)	48.6	43.7	50.7	0.279
≥30	384 (41)	59	58.8	64	0.03

Values in parentheses are percentages. *Log-rank test.

**Table 5:** Significant prognostic factors evaluated by Cox proportional hazards analysis

	**Overall survival**	
	**Hazard ratio**	***P***
**Tumor location**		
Lower third	0.54 (0.26-1.11)	0.095
Middle third	0.53 (0.25-1.09)	0.08
Upper third	0.91 (0.43-1.9)	0.8
Whole stomach	1	
**Tumor stage**		
I	0.15 (0.08-0.28)	<0.001
II	0.35 (0.2-0.62)	<0.001
III	0.69 (0.41-1.15)	0.16
IV	1	
**Treatment period**		
1980-1989	1.25 (0.95-1.65)	0.1
1990-1999	1.02 (0.76-1.36)	0.86
2000-2010	1	
**Lauren classification**		
Diffuse	1.34 (0.86-1.73)	0.78
Indeterminate	1.52 (1.2-1.81)	0.41
Intestinal	1	
**Lymph nodes retrieved**		
<15	2.41 (1.07-2.86)	
15-30	1.34 (1.02-2.15)	0.009
≥30	1	
**Patients with complications**		
Yes	1.19 (0.93-1.5)	0.156
No	1	
**Multivisceral resections**		
Yes	1.07 (0.81-1.43)	0.609
No	1	
**Margin status**		
R1/R2	1.97 (1.2-3.1)	0.003
R0	1	

Values in parentheses are 95% confidence intervals.

The overall median survival was 52 months. The median survival in the three time periods was 37, 54, and 68 months, respectively.

**Figure 1:**
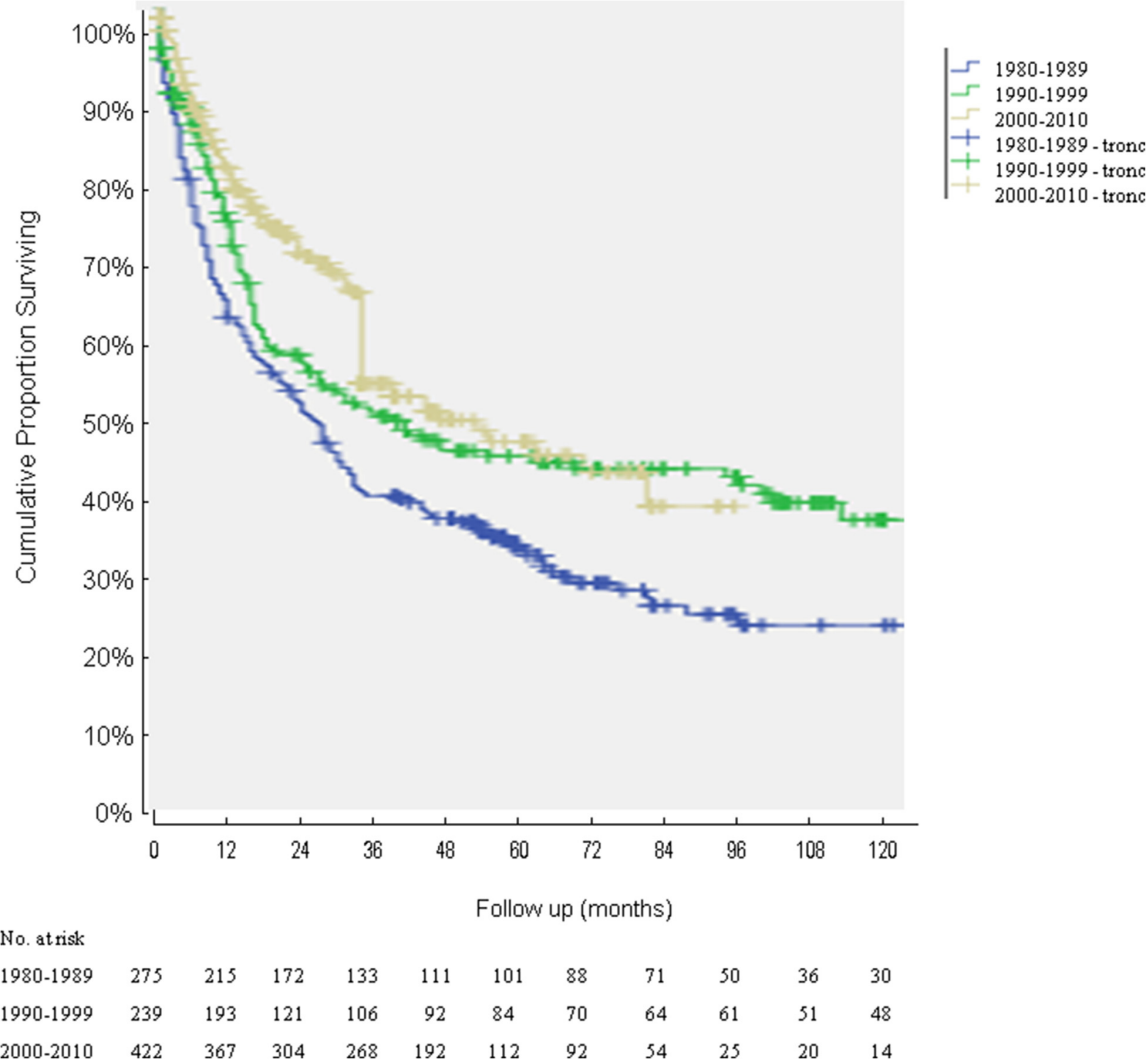
**Overall survival curves calculated by the Kaplan-Meier method for the three treatment periods; 936 resected patients were included; *P <*** **0.001 (period 1980-1989 versus 1990-1999/2000-2010; log rank).**

The five-year overall survival for the three periods was 39%, 48%, and 51%, respectively (period 1980-1989 versus 1990-1999/2000-2010) *P <*0.001; ten-year overall survival for the three periods was 27%, 42%, and 42%, respectively, *P <*0.001 (period 1980-1989 versus 1990-1999/2000-2010). See Figure 1.

**Figure 2:**
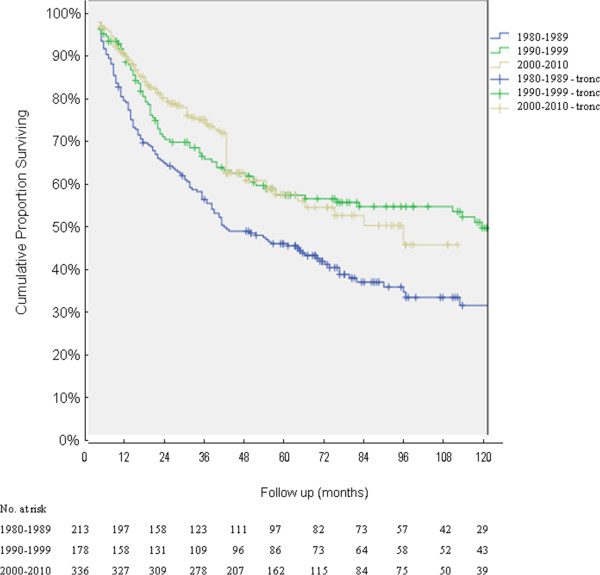
**Overall survival curves calculated by the Kaplan-Meier method for R0 for the three treatment periods; 723 resected patients were included; *P <*** **0.001 (period 1980-1989 versus 1990-1999/2000-2010; log rank).**

Over time there was an improvement in the R0 five-year survival rate: the survival significantly increased from 51% to 64.5% (*P* < 0.001). See Figure 2.

Survival was strongly associated with tumor location, tumor stage, R-classification, and lymph node dissection at univariate analysis.

For stage III cancer there was a 10% increase (from 28 to 38%; *P =* 0.024). A significant increase of five-year survival was encountered for patients with less than 15 lymph nodes retrieved (from 22.4 to 39.2%; *P* < 0.001) as well.

In multivariate analysis, tumor stage, lymphadenectomy, and margin status were independent prognostic indicators.

## Discussion

In this large Western single institution series, GC characteristics, surgical approaches, and long-term results were analyzed over a period of 30 years in a tertiary care hospital.

Throughout the world, stomach cancer is a disease of the elderly population with a predominance in men [15].

The mean age and male predominance of the patients in our series are similar to those in other reports [13,15,16].

Trend analyses in our study showed no changes in mean age or gender proportions over the years.

Usually, postoperative outcomes in Western centers have been explained by the difference in age, weight, and comorbidity, whereas the poor long-term survival has been attributed to tumor stage and location [16,17].

In contrast with other reports in the literature [17-21], in our experience we observed a constant increase of middle third location with respect to upper third location. The significant increase, between the second and the third decade, in subtotal distal gastrectomies may be partially explained by a different attitude to perform a partial gastrectomy rather than a total gastrectomy in the case of a middle third location.

The highly significant difference in tumor stage between time periods is undoubtedly related to the widespread use of endoscopy in case of gastric symptoms, which resulted in earlier diagnosis.

Over time, we assisted to a significant decrease in perioperative mortality and overall complications; *P* = 0.009 and *P* = 0.004, respectively.

Anesthesiological improvements have significantly reduced perioperative death to a minimum, and support is readily available in the intensive care unit if postoperative organ failure occurs. Besides these general improvements in perioperative care, the refinement of the surgical technique may also have contributed to improve early results after gastrectomy. The introduction of the pancreas-preserving procedure [22], the avoidance of splenectomy if unnecessary, and the evolution of anastomotic techniques have reduced the incidence of postoperative surgical complications [23,24].

Over the three decades, survival for the present study was significantly better with respect to other Western experiences [17,18,21].

In this study, we compared survival rates of patients with resected GC after stratification by time periods. Our results largely agree much more with Eastern series [25] than with previously reported Western experiences [26-28].

Karpeh *et al.*[19], for example, reported the overall fve-year survival rate after R0 resection for GC to be 49%.

Marrelli *et al.*[18], in a large multicenter Italian observational study, had concluded that five-year survival rates after R0 resection (2,320 patients) did not change over time; moreover, a mild decrease in survival in more recent years was related to more advanced stages, distal tumors, and tumors in women. Marrelli *et al.* reported a five-year overall survival rate of 54.7%; 51.2% in the most recent period.

In a large meta-analysis of 100 English-language publications since 1970, Akoh and Macintyre [29] found survival to improve from early to later time periods.

In the current study, we found a significant improvement in survival among the three study periods (*P* < 0.001) comparing period 1 versus periods 2 and 3.

First of all, this improvement is undoubtedly related to the increased number of early gastric cancers due to the widespread use of endoscopy in the case of gastric symptoms. Moreover, the increase in long-term survival after curative gastrectomy is probably related to our maximum effort to effect a surgical cure. N and R variables were addressed by routine extended lymphadenectomy. R0 resection was obtained in 77.2% of patients, and most of them received an extended lymphadenectomy.

The mean number of dissected nodes per operative specimen was high. Appropriate training in the techniques of extended lymphadenectomy is essential. The clearance of appropriate lymph node stations was not compromised by case difficulty, and accurate staging and optimization by a multidisciplinary team are essential. Appropriate management of postoperative complications is crucial to minimize mortality. A meta-analysis of six randomized controlled trials totalling 1,876 patients concluded that D2 gastrectomy is associated with higher 30-day mortality and more postoperative complications and with a five-year survival similar to that of the D1 cohort [30].

However, experienced groups from Japanese [31,32] and Western [13,33,34] institutions continue to perform complete D2 lymph node dissection, reporting low complication rates and survival advantages. Similar results have been obtained in recently published prospective studies [35,36].

Extended lymphadenectomy leads to improved long-term survival without compromising postoperative outcomes.

Multivariate analysis revealed a strict correlation between the number of lymph nodes removed and survival advantages; therefore, we can conclude that the number of lymph nodes dissected is significantly indicative of the quality of surgery [37].

Conversely, the constant increase in survival for patients with stage III cancer and for those with fewer than 15 lymph nodes removed is probably due to the positive effects of chemotherapy regimens based on epirubicin, cisplatin, and fluorouracil adopted in our center.

With respect to the Lauren classification of tumor type, at univariate analysis we found that the survival of patients with diffuse-type histology was significantly lower than that of patients with intestinal-type histology, but only in the third decade. This is consistent with published studies that do not exclude EGJ tumors from the analysis [38]. It is an important consideration, because tumors in the EGJ are more likely to be of the diffuse-type histology with a correspondingly poorer prognosis.

In the present series, we note a steady increase in survival, over time, both for diffuse-type and intestinal-type histology, *P* = 0.003 and *P* = 0.017, respectively.

Regarding the association between tumor location and survival, conflicting data exist. Studies that eliminate EGJ tumors, for example, find no significant association between survival and tumor location [38,39].

However, other studies that included EGJ tumors in the analysis have found that distal tumors are associated with an improved prognosis compared with proximal tumors [40,41]. The major determinants of the poor prognosis of proximal GC with respect to distal GC rely both on the more advanced age and tumor stage at the moment of clinical presentation and on the higher postoperative morbidity for patients with proximal GC.

Our series seems to confirm this latter position, excluding from the analysis EGJ tumors.

The constant increase of multivisceral resections in our series can be explained by the more aggressive surgical approach in the case of locally advanced GC and in the suspicion of direct invasion of adjacent organs. As reported by a recent Italian multicenter observational study [42], patients undergoing extended resections experience acceptable postoperative morbidity and mortality rates, and an en bloc multivisceral resection should be performed in patients when a complete resection can be realistically obtained and when lymph node metastasis is not evident.

The main limitations of this study include the following: 1) outcomes are from a single institution; 2) the study has a retrospective nature, which precludes the controlled acquisition and real-time verification of data that benefit prospective series; 3) follow-up is incomplete (less than five years) for a few patients of the last decade; 4) neoadjuvant and adjuvant treatment data are heterogeneous; and 5) quality-of-life data are absent.

In our retrospective study, we observed no significant improvement in survival with adjuvant therapy, likely due to the lack of a uniform regimen over the study’s 30-year period. In any case, the potential benefit of postoperative adjuvant chemotherapy is still unclear [43].

Twenty-eight patients who received neoadjuvant chemotherapy according to the MAGIC protocol were not taken into consideration for statistical analysis because they were all included in the last decade.

Significant survival improvement with perioperative therapies has been reported, and this treatment may be particularly useful for tumors with serosal involvement or advanced lymph node spread [14].

## Conclusions

In conclusion, in our experience in the treatment of GC patients over three decades, we have observed a significant improvement in perioperative and postoperative care and a steady increase in overall survival.

Promising results are expected by the constant use of neoadjuvant regimens for locally advanced diseases.

Moreover, in the near future, we might expect some improvements in terms of quality of life from the increasingly widespread use of endoscopic dissection for early GC and minimally invasive surgical therapies.

## Competing interests

The authors declare that they have no competing interests.

## Authors’ contributions

FR drafted the manuscript and searched the literature. SA, GBD and GC participated in the design of this study and critically revised the manuscript for important intellectual content. APT and CF performed the statistical analysis and collected important background information. All authors read and approved the final manuscript.
